# Alert for COVID-19 second wave: A lesson from Vietnam

**DOI:** 10.7189/jogh.11.03012

**Published:** 2021-01-16

**Authors:** Thanh Ha Le, Thi Phuong Thao Tran

**Affiliations:** 1 Graduate School of Cancer Science and Policy, National Cancer Center, Goyang-si, Republic of Korea; 2 Center for Population Health Sciences, Hanoi University of Public Health, Hanoi, Vietnam

**Figure 1:**
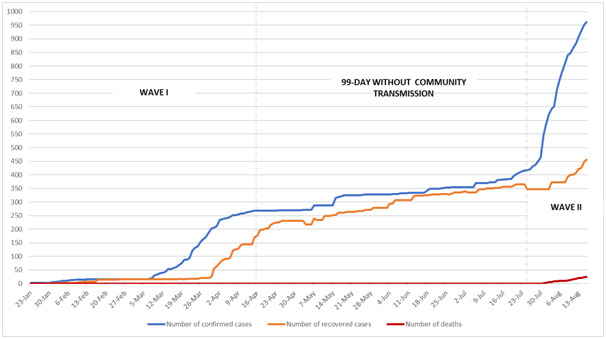
The cumulative number of COVID-19 confirmed cases, recovered cases and number of deaths in Vietnam.

The COVID-19 pandemic has posted an unprecedented new threat as many countries are seeing a resurgence of the disease after successfully controlling outbreaks early in the year. As China eases some stringent coronavirus-related restrictions after months with sporadic cases, its capital has raised the dangerous level as the total number of infections to 137 in one week in mid-June, the highest daily confirmed cases since March [1]. After the first COVID-19 curve flattening, Japan has entered the second wave with 12 629 people being treated in hospital as of 6 August, warning that an emergency may be declared for some cities since the nationwide month-long state of emergency ended in May [2]. Specifically, coronavirus has re-emerged in some Asia countries previously known as containment models such as Hong Kong, Australia, and so forth. In line with other countries, Vietnam is now also struggling with the second wave of the COVID-19 (**Figure1**).

Vietnam’s first case was reported on January 23, marking a fierce period against COVID-19 in Vietnam. A series of drastic and urgent measures, such as border closure, restrictions on domestic and international movements, school and workplace shutdown, cancellation of public events and gatherings, strict quarantine, social distancing, and effective communication strategies have been imposed to minimize the spread of virus in community and from other affected areas [3]. These decisive and rapid efforts kept the number of cases low at 268, allowing Vietnam to get into 99 consecutive days without community transmission since April 16 [4]. During the 99 days, life was gradually returning to a kind that has been missing for months. School reopened to welcome students after more than 3-absence-month since Vietnam Lunar New Year. All economic activities such as restaurants, shops, and public services were able to operate again. Notably, domestic travel activities are getting busy again in many coastal tourism localities thanks to promoted travel packages. From April 19 to May 18, 2020, the number of flights by local carriers was 8623, which increased 73.7% compared to previous month, especially in Da Nang, one of the best tourist destinations, to where 454 764 travellers visited in June [5]. All new confirmed cases in these 99 days were Vietnamese citizens who returned from abroad and quarantined upon arrival without any risk of community transmission, raising the total number of cases from 268 to 415 cases [4].

The country is bracing for the second wave of COVID-19 infection after recording a 57-year-old man with an unknown source of infection in Da Nang C hospital, ending its 99-day streak without community transmission. Vietnamese scientists have revealed that this type is the new, more contagious virus strain, which has been seen in Bangladesh, Britain, and Ireland. This strongly suggests that the new case was introduced from outside Vietnam [6]. In the next few days, the clusters quickly emerged in three hospitals in Da Nang. All new COVID-19 cases related to Da Nang C hospital and they had contacts with suspected patients. Furthermore, Da Nang is a popular tourist hotspot, making thousands of travellers’ possible sources of virus infection throughout the country. As predicted, during nearly one month, the total of confirmed cases doubled from 416 to 962 and widely spread to 15 cities/provinces from North to South as of August 16 [4]. Vietnam recorded its first-ever death on July 31, which rapidly increased to 21 deaths by August 15. The mortality increased steeply because almost all the deceased were inpatients with adverse underlying medical conditions who were being treated in hospitals in Da Nang. To respond to such emergencies, the government ordered Da Nang authorities to halt all charter flights to Da Nang international airport from the very first time after the case was recognized. In late July, Da Nang strictly placed seven districts in quarantine and rapidly imposed a lockdown in four hospitals including Da Nang C hospital, Da Nang Hospital, Da Nang Orthopedic and Rehabilitation Hospital, and Hoan My Hospital. Da Nang has started implementing social distancing, requesting people not go out unless for necessities such as buying food or drugs. In the capital Hanoi and other provinces, contact tracing and mass testing have been conducted extensively for people who recently returned from Da Nang to suppress the outbreak. On August 5th, one temporary field hospital has been built inside the largest sport center in Da Nang in anticipation of rising numbers of COVID-19 patients. Hundreds of doctors, nurses, and medical students have been mobilized to combat the COVID-19 in its epicenter – Da Nang.

**Figure:**
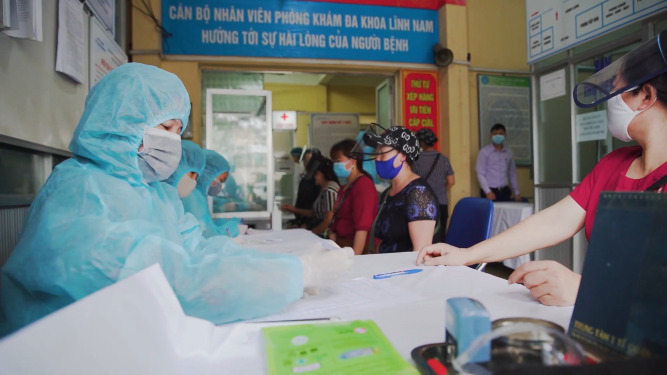
Photo: Vietnamese people registered for rapid testing (from: https://commons.wikimedia.org/wiki/File:Vietnamese_registered_for_rapid_testing_(COVID-19).png).

The question raised here is where the outbreak originated while Vietnam strictly implemented border shutdown, suspended international flights, and all arrivals in mandatory 14-day quarantine facilities? The unidentified source of infection of the first case in Da Nang (in the second wave) could originate from the outside, as illegal foreigners may have entered Vietnam [2]. This was strongly confirmed when a Chinese who illegally entered Ho Chi Minh city tested positive for SARS-CoV-2 [7]. In fact, neighbouring countries of Vietnam including China, Cambodia and Laos have still tackled complicated developments of the pandemic. Meanwhile, hundreds of foreigners have illegally entered Da Nang and Ho Chi Minh City by road, and they were detected in July [2]. In particular, 27 Chinese citizens were arrested when deliberately immigrating to Da Nang in mid-July. Da Nang’s police have arrested one person and prosecuted three people, including one Chinese and two Vietnamese, for organizing illegal entry into Vietnam for foreigners. In Nghe An province, central part of Vietnam, the border force also arrested 3 people who illegally crossed into Vietnam from Laos. In the South, An Giang province arrested 41 people traveling from Cambodia to Vietnam by sea in eight small boats.

Most of Vietnam’s boundaries consist of jungle or rugged mountains, which significantly hinders government immigration management. Consequently, unauthorized immigration is a loophole in preventing and combating the outbreak. In other countries like Malaysia, authorities arrested 586 undocumented migrants [8]. They did this on May 2, to prevent the potential outbreak. In Thailand, the head of Thai immigration office said they were also concerned about ongoing and illegal crossing among workers who may bring viral infections that could threaten Thailand’s success in combating COVID-19 [9].

A series of emergency restrictions were imposed to prevent illegal entry. The COVID-19 National Steering Committee Meeting immediately enacted the Official dispatch No.3961/CV-BCĐ imposing tighter border controls to crack down on illegal immigrants from neighbouring countries on July 25 [10]. Government asked residents to report to the local authorities on any suspected or unlawful entry for testing and quarantine. In just over six months, Vietnam has established nearly 1600 border checkpoints with more than 9400 active residents identifying illegal immigrants, imposing infection control measures upon them. All acts of assisting in or covering for undocumented immigration and illegal entrants would face stiff penalties according to current regulations.

Although it is still unknown where the outbreak originating from, illegal immigration remains a pressing problem in Vietnam, prompting public concern about the spread of COVID-19 and other infectious diseases. In the initial period of the pandemic, Vietnam was praised globally for its prompt and decisive measures to combat the outbreak. Nevertheless, the country has been placed on an alert again, facing unprecedented new circumstances. As long as this uncertainty remains, the critical lesson about the resurgence outbreak in Vietnam – related to illegal entry activities – might be a reminder for other countries on the options that the governments should consider to proactively manage the potential risks of COVID-19 pandemic.
